# Supplementary material for: Olfactory screening of Parkinson’s Disease patients and healthy subjects in China and Germany: A study of cross-cultural adaptation of the Sniffin’ Sticks 12-identification test
Source: PLoS One. 2019 Nov 8;14(11):e0224331. doi: 10.1371/journal.pone.0224331 (PMC6839844; doi:10.1371/journal.pone.0224331)
Supplement: Attachment — Submitted filename: PLOS PONE Pinkhardt _review_260619.docx [file pone.0224331.s002.docx]

Review

PLPS one manuscrpit PONE D 19 15889

By inkhardt E et al.

This manuscript reports the first study to directly compare olfactory function in a North Chinese and a German cohort of healthy subjects and Parkinson patients. For testing olfactory function the SIT12 (Sniffin Sticks) test and a modified CH-SIT12 test – the later adapted to the Chinese culture – are employed.

The study is explanatory and therefore does not fulfill the strict criteria of a test-retest development with all the statistical ramifications. By testing all 16 odors in one test procdur, the need for adjusting the analysis for multiple testing was avoided.

There are a few comments:

Line 86 is not clear – cited: for the patient groups we likewise hypothesize …… please change

Line 119: was the UPDR III rating performed in On or Off – I assume the PD patients were under treatment.

Line 167: Is there evidence that olfactory function deteriorates during the progression of PD or is the hyposmia stable over time ?

Please quote respective references.

Table 1

20 % of German PD patients were smokers. Did they perform differently ?

Were the Chinese PD patients de novo patients ? They had a disease duration of about 3 years.

Was the Sniffin Test performed by asking the person to sniff once or twice ? – there is a difference in the results if you sniff once or twice

Line 191-199: For a naive reader this paragraph is slightly confusing. Please try to present the data in a simpler way.

Discussion:

Line 312: The most likely explanation of the results is the fact that German subjets are widely exposed to Chinese Cuisine.

It is not surprising that liquorice and coffee are well recognized by the Chinese Healthy controls and PD patients. This is part of their day to day diet . Thus it is not clear why these two odors where exchanged. This should be explained in the methods.

Conclusion: it may be changed. As in the strict sense the CH-SIT12 would only need an exchange of 2 odors and a replacement for the oder shoeleather.

Criteria for publications:

Original

Trans-national

Nicely carried out

Minor modifications requested.

Accept for publication.
